# Alcopops, taxation and harm: a segmented time series analysis of emergency department presentations

**DOI:** 10.1186/s12889-015-1769-3

**Published:** 2015-05-06

**Authors:** Marianne Gale, David J Muscatello, Michael Dinh, Joshua Byrnes, Anthony Shakeshaft, Andrew Hayen, Chandini Raina MacIntyre, Paul Haber, Michelle Cretikos, Patricia Morton

**Affiliations:** Public Health Officer Training Program, New South Wales Ministry of Health and School of Public Health and Community Medicine, University of New South Wales, 73 Miller Street, North Sydney, NSW 2060 Australia; New South Wales Ministry of Health and School of Public Health and Community Medicine, University of New South Wales, 73 Miller Street, North Sydney, NSW 2060 Australia; Royal Prince Alfred Hospital and Sydney Medical School, University of Sydney, Missenden Road, Camperdown, NSW 2050 Australia; Centre for Applied Health Economics School of Medicine, Griffith University, Meadowbrook, QLD 4131 Australia; National Drug and Alcohol Research Centre, University of New South Wales, Sydney, NSW 2052 Australia; School of Public Health and Community Medicine, University of New South Wales, Sydney, NSW 2052 Australia; New South Wales Ministry of Health, 73 Miller Street, North Sydney, NSW 2060 Australia

**Keywords:** Alcohol harm, Alcopops, ‘Ready-to-drink alcohol’, RTDs, Alcohol taxation, Emergency department presentations

## Abstract

**Background:**

In Australia, a Goods and Services Tax (GST) introduced in 2000 led to a decline in the price of ready-to-drink (RTD) beverages relative to other alcohol products. The 2008 RTD (“alcopops”) tax increased RTD prices. The objective of this study was to estimate the change in incidence of Emergency Department (ED) presentations for acute alcohol problems associated with each tax.

**Methods:**

Segmented regression analyses were performed on age and sex-specific time series of monthly presentation rates for acute alcohol problems to 39 hospital emergency departments across New South Wales, Australia over 15 years, 1997 to 2011. Indicator variables represented the introduction of each tax. Retail liquor turnover controlled for large-scale economic factors such as the global financial crisis that may have influenced demand. Under-age (15–17 years) and legal age (18 years and over) drinkers were included.

**Results:**

The GST was associated with a statistically significant increase in ED presentations for acute alcohol problems among 18–24 year old females (0 · 14/100 000/month, 95% CI 0 · 05-0 · 22). The subsequent alcopops tax was associated with a statistically significant decrease in males 15–50 years, and females 15–65 years, particularly in 18–24 year old females (−0 · 37/100 000/month, 95% CI −0 · 45 to −0 · 29). An increase in retail turnover of liquor was positively and statistically significantly associated with ED presentations for acute alcohol problems across all age and sex strata.

**Conclusions:**

Reduced tax on RTDs was associated with increasing ED presentations for acute alcohol problems among young women. The alcopops tax was associated with declining presentations in young to middle-aged persons of both sexes, including under-age drinkers.

## Background

Alcohol causes a significant burden of disease in Australia with around 5000 deaths and 157 000 hospitalisations attributable to alcohol each year [1]. In New South Wales (NSW) 41% of males and 34% of females aged 16–24 report drinking at levels that pose a lifetime risk to their health. Compared to the general population, young Australians are twice as likely to drink at very high levels on a single occasion [2].

Reducing alcohol-related harms in young people is a major public health goal in Australia and a pressing political issue. Recent economic analyses have shown that taxation leading to increased alcohol price for consumers would be more cost effective in reducing alcohol related harm in Australia than a range of other interventions [3] and that increasing price is likely to impact more on young drinkers than older drinkers as young people are more price sensitive [4].

In April 2008 the Australian Federal Government imposed a 70% tax increase on ‘ready to drink’ alcoholic beverages (RTDs, pre-mixed drinks, or “alcopops”) from $AUD39 · 36 to $AUD66 · 67 per litre of alcohol bringing the excise on RTDs into line with the excise on straight spirits. This policy intervention, known as the alcopops tax, aimed to reduce harm from binge drinking among young people, especially females who are the primary target for RTDs which have packaging and marketing strategies specifically designed to attract youth [5]. The mixing of alcohol with familiar sweet tastes such as cola or fruit juices has been shown to improve the appeal of alcoholic drinks to young people, facilitate premature consumption of alcohol and encourage increased volume of alcohol consumption [6].

The alcopops tax was introduced in response to concerns about the explosive growth in sales and consumption of RTDs that had occurred in Australia since July 2000 when tax reform involving the introduction of a Goods and Services Tax (GST) left RTDs taxed at 40% less per litre of alcohol compared with straight spirits. As RTD producers exploited this tax advantage, prices were driven further down between 2000 and 2007 rendering RTDs a substantially cheaper drink choice relative to other spirits. During this period, RTDs arguably not only competed for market share, but also increased the overall alcohol market in Australia [7].

In the two years following the introduction of the alcopops tax, sales data showed a decline in consumption of RTDs [8,9]. However, no studies have examined the longer term impact of the tax on alcohol consumption, and limited evidence has emerged as to whether this specific taxation measure reduced rates of alcohol-related harm in young people as was intended.

To address this evidence gap, this study estimates the impact of changes in alcohol taxation on presentations for acute alcohol problems to hospital Emergency Departments (EDs) in NSW.

## Methods

### Data sources

The Emergency Department Data Collection (EDDC) of the NSW Ministry of Health is the largest database of ED presentations in Australia. Public hospital EDs were included in the analysis if they (i) reported continuously during the 15 year study period 1 January 1997 to 31 December 2011, and (ii) had greater than 90% completeness of diagnostic information recorded over the same period. 39 EDs met the criteria. Of these, 24 (62%) were located in the metropolitan Sydney area. The included EDs accounted for approximately 57% of all public hospital ED presentations in NSW.

Time series of monthly counts of ED presentations assigned a provisional diagnosis of acute alcohol problems were assembled and converted to age and sex-specific population rates. As there are no defined population catchments for NSW hospitals, estimated NSW resident populations from the Australian Bureau of Statistics (ABS) were used as denominators. Series were prepared for each sex in the age groups: 15–17,18-24, 25–49, 50–64 and 65 years and over.

Due to variation in hospital ED coding systems in NSW, we selected provisional diagnoses for acute alcohol problems using the International Classification of Diseases (ICD), ninth revision (Australian clinical modification or ICD-9-CM) codes: 291, E860, 303, 305 · 0, 790 · 3, 980, V70 · 4; the ICD-10 (Australian clinical modification or ICD-10-AM) codes F10, R78 · 0, T51, X45, X65, Y15, Y90-91, Z04 · 0, Z72 · 1 or Z86 · 41; and related Systematized Nomenclature of Medicine, Clinical Terminology (SNOMED-CT) concept identifiers (available on request). The codes relate to: alcohol intoxication, poisoning, dependence, withdrawal, elevated blood alcohol reading, and medico-legal blood alcohol or drug test.

Data on the monthly retail turnover of liquor in NSW was obtained from the ABS [10]. Original, non-seasonally adjusted values were used, to be consistent with the ED time series. Per capita estimates of retail turnover of liquor were calculated using NSW estimated resident populations in persons aged 18 years and over. The Consumer Price Index (CPI) for alcohol in Sydney was used to adjust for inflation [11].

To assist interpretation of the findings, national apparent consumption of alcohol trends by drink type was graphed [12].

### Statistical analysis

A segmented time series regression analysis was performed for the whole sample and repeated for each of the ten age and sex strata. Monthly population rates of ED presentations for acute alcohol problems were the dependent variables.

Two indicator variables were included, one for the introduction of the GST on 1 July 2000, and the other for the introduction of the alcopops tax on 27 April 2008. The indicator variables were defined as zero for each month prior to the tax change it represented, and unity (1) for each remaining month in the study period.

For each tax, another variable was created with a value of zero prior to the intervention, and counting from 1 for each month from the beginning of the tax intervention. These variables are equivalent to an interaction between ‘month’ and each tax indicator variable. This allows estimation of tax associated slope changes in the ED time series [13].

Monthly retail liquor turnover in NSW was included to control for economic factors, such as the global economic decline following the Global Financial Crisis (GFC) of 2007, which may have been an important confounder in the study.

Ordinary least squares regression assuming normally distributed errors was used. Quantile-quantile (Q-Q) plots for assessing normality in the residuals of each model were checked. The Durbin-Watson Statistic and autocorrelation charts were used to assess the assumption of independence and lack of higher order autocorrelation lagged up to 24 months in model residuals.

In case of incomplete control of the effects of the GFC, a sensitivity analysis was performed adding a time series of per capita Gross Domestic Product (GDP) into the model. Non-identified data, as defined by the Australian National Health and Medical Research Council’s National Statement on Ethical Conduct in Human Research was used for this study and ethics approval was not required. Data analysis was conducted with the REG procedure using SAS version 9.3 through SAS Enterprise Guide version 5 · 1.

## Results

### Descriptive statistics

From 1 January 1997 to 31 December 2011, there was a total of 107 810 presentations for acute alcohol problems to the 39 emergency departments. Of these, 70 740 (66%) were by males and 29 265 (27%) were to rural or remote hospitals. Of the 20 523 692 total ED presentations during the period, 97% had complete diagnostic information recorded.

**Figure 1:**
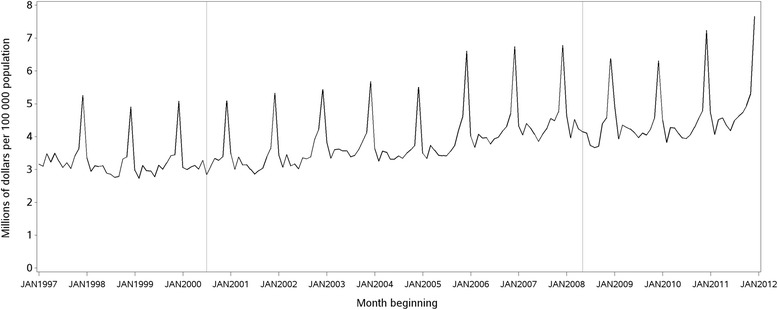
Retail turnover of liquor per 100 000 population aged 18 years and over, NSW, 1997–2011. Retail turnover of liquor is adjusted for the consumer price index, and expressed in millions of Australian dollars (AUD).

The overall median monthly rate of presentations for acute alcohol problems was 10.5/100 000 and the highest sex/age strata were 18–24 year old males and females (18 · 9 and 18 · 3/100 000 respectively) (Table 1). The median monthly retail turnover of liquor in NSW was 3 · 7 million Australians Dollars (inter-quartile range = 1 · 0) per 100 000 population 18 years and over. The time series for retail turnover of liquor in NSW from 1997 to 2011 is illustrated in Figure 1.

**Table 1:** **Summary statistics for the total monthly population rate of emergency department presentations for acute alcohol problems to 39 NSW hospitals, by age and sex, 1997–2011**

**Age group**	**Sex**	**Median rate/100 000 population**	**Lower quartile of rate/100 000 population**	**Upper quartile of rate/100 000 population**
Overall		10 · 5	9 · 1	12 · 8
15-17 years	Female	15 · 6	11 · 8	20 · 2
	Male	14 · 3	10 · 9	19 · 4
18-24 years	Female	18 · 3	10 · 9	26 · 2
	Male	18 · 9	15 · 2	26 · 8
25-49 years	Female	6 · 9	5 · 3	9 · 1
	Male	16 · 1	14 · 4	18 · 2
50-64 years	Female	4 · 3	3 · 3	5 · 7
	Male	13 · 1	11 · 5	15 · 2
65 years and over	Female	1 · 7	1 · 2	2 · 1
	Male	6 · 5	5 · 5	7 · 6

### Segmented time series regression models

Q-Q and autocorrelation plots and the Durbin-Watson statistic showed that the model residuals conformed to assumptions of independence and normality.

Following the GST, a statistically significant increase in the slope of ED presentation rates for acute alcohol problems was seen overall, and in 18–24 year old females (0 · 14 ED presentations/100 000/month, 95%CI 0 · 05-0 · 22). There was no significant change in the slope of the rate of ED presentations for any other sex/age group. There was also a statistically significant level decrease overall and in 18–24 year old females (Table 2). The level change reflects a constant shift across the entire duration of the intervention period and does not accumulate over time.

**Table 2:** **Association between introduction of the Goods and Services Tax in July 2000 and the total rate of emergency department presentations for acute alcohol problems to 39 NSW hospitals, by age and sex, 1997-2011**

**Age group**	**Sex**	**Level change/100 000 population (95% CI)**	**Slope change/100 000/month (95% CI)**
Overall		−0 · 87 (−1 · 40 to −0 · 35)	0 · 046 (0 · 037 to 0 · 054)
15-17 years	Female	−0 · 46 (−3 · 73 to 2 · 82)	0 · 08 (−0 · 04 to 0 · 20)
	Male	−2 · 30 (−5 · 18 to 0 · 58)	0 · 02 (−0 · 09 to 0 · 12)
18-24 years	Female	−2 · 53 (−4 · 87 to −0 · 19)	0 · 14 (0 · 05 to 0 · 22)
	Male	−3 · 48 (−5 · 84 to −1 · 13)	−0 · 01 (−0 · 09 to 0 · 08)
25-49 years	Female	−0 · 77 (−1 · 53 to −0 · 005)	0 · 02 (−0 · 01 to 0 · 04)
	Male	−2 · 56 (−3 · 88 to −1 · 24)	0 · 03 (−0 · 02 to 0 · 08)
50-64 years	Female	−0 · 41 (−1 · 21 to 0 · 39)	0 · 004 (−0 · 03 to 0 · 02)
	Male	−1 · 87 (−3 · 35 to −0 · 39)	−0 · 05 (−0 · 10 to 0 · 003)
65 years and over	Female	−0 · 13 (−0 · 58 to 0 · 31)	−0 · 0003 (−0 · 017 to 0 · 016)
	Male	−0 · 56 (−1 · 62 to 0 · 49)	0 · 01 (−0 · 02 to 0 · 05)

CI – confidence interval.

Following the alcopops tax, statistically significant decreases in the slope of ED presentation rates for acute alcohol problems were seen overall, and for males and females in the age groups 15–17, 18–24 and 25–49 as well as for females 50–64 years. The greatest decreases in slope were seen in 18–24 year old females (−0 · 37 ED presentations/100 000/month, 95% CI −0 · 45 to −0 · 29) followed by 18–24 year old males. Following the alcopops tax, there was also a statistically significant constant level increase in the rate of acute alcohol-related ED presentations among males aged 15–17 years and 18–24 years, and females aged 50–64 years (Table 3).

**Table 3:** **Association between introduction of the alcopops tax in April 2008 and total rate of ED presentations for acute alcohol problems to 39 NSW hospitals, by age and sex, 1997-2011**

**Age group**	**Sex**	**Level change/100 000 population (95% CI)**	**Slope change/100 000/month (95% CI)**
Overall		0 · 60 (−0 · 16 to 1 · 35)	−0 · 10 (−0 · 12 to −0 · 07)
15-17 years	Female	2 · 26 (−1 · 06 to 5 · 59)	−0 · 24 (−0 · 35 to - 0 · 12)
	Male	3 · 91 (0 · 98 to 6 · 82)	−0 · 20 (−0 · 29 to −0 · 10)
18-24 years	Female	−0 · 70 (−3 · 07 to 1 · 68)	−0 · 37 (−0 · 45 to −0 · 29)
	Male	2 · 64 (0 · 25 to 5 · 03)	−0 · 26 (−0 · 34 to −0 · 18)
25-49 years	Female	0 · 49 (−0 · 29 to 1 · 26)	−0 · 08 (−0 · 10 to −0 · 05)
	Male	0 · 30 (−1 · 04 to 1 · 64)	−0 · 08 (−0 · 13 to −0 · 04)
50-64 years	Female	1 · 22 (0 · 41 to 2 · 03)	−0 · 05 (−0 · 08 to −0 · 02)
	Male	−0 · 58 (−2 · 08 to 0 · 92)	−0 · 03 (−0 · 08 to 0 · 02)
65 years and over	Female	0 · 05 (−0 · 40 to 0 · 51)	−0 · 007 (−0 · 02 to 0 · 008)
	Male	0 · 69 (−0 · 38 to 1 · 77)	−0 · 02 (−0 · 06 to 0 · 02)

CI – confidence interval.

Retail liquor turnover showed a positive and statistically significant association with ED presentations for acute alcohol problems overall and in all sex/age strata. The strongest associations were for males aged 15–17 and 18–24 years (Table 4).

**Table 4:** **Association between total monthly retail turnover of liquor in NSW and total monthly rate of ED presentations for acute alcohol problems to 39 NSW hospitals, by age and sex, 1997-2011**

**Age group**	**Sex**	**Change/100 000/$AUD 1 million (95% CI)**
Overall		1 · 18 (0 · 97 to 1 · 41)
15-17 years	Female	1 · 15 (0 · 16 to 2 · 13)
	Male	2 · 68 (1 · 82 to 3 · 54)
18-24 years	Female	1 · 78 (1 · 08 to 2 · 48)
	Male	2 · 49 (1 · 78 to 3 · 19)
25-49 years	Female	0 · 81 (0 · 59 to 1 · 04)
	Male	1 · 88 (1 · 48 to 2 · 27)
50-64 years	Female	0 · 56 (0 · 33 to0 · 80)
	Male	1 · 17 (0 · 72 to 1 · 61)
65 years and over	Female	0 · 23 (0 · 09 to 0 · 36)
	Male	0 · 47 (0 · 16 to 0 · 79)

CI – confidence interval.

AUD – Australian Dollars.

The GDP time series was strongly correlated with retail turnover of liquor. Sensitivity analysis including GDP did not alter our conclusions in a practically significant way. Therefore GDP was excluded from the final models.

**Figure 2:**
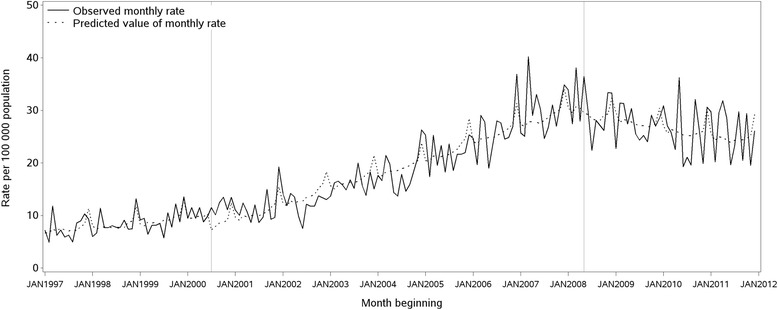
Total monthly rate of ED presentations for acute alcohol problems, females 18–24 years, 1997–2011.

**Figure 3:**
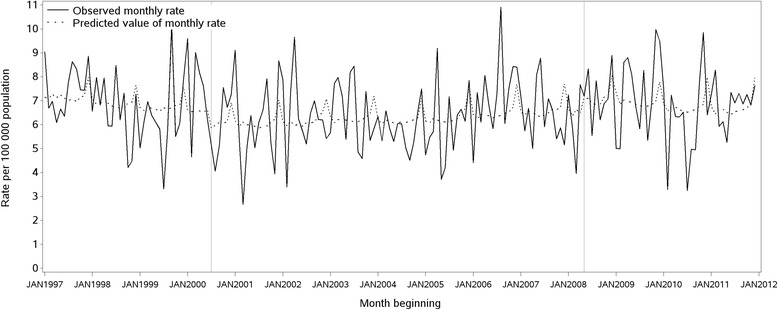
Total monthly rate of ED presentations for acute alcohol problems, males 65 years and over, 1997–2011.

**Figure 4:**
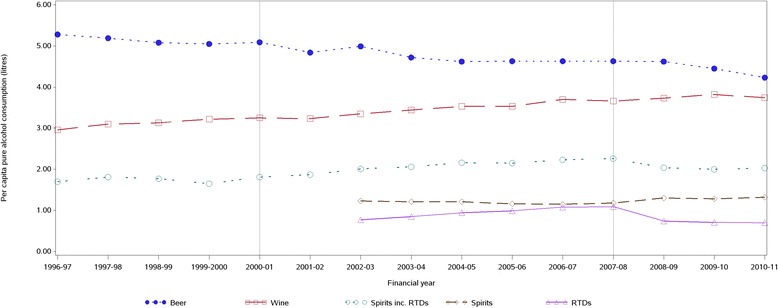
Annual per capita consumption of beer, wine, spirits and RTDs in Australia, 1996–2011. Source: Australia Bureau of Statistics. Apparent Consumption of Alcohol, Australia 2010–2011.

Examples of regression models that demonstrated strong and weak correlations are illustrated in Figure 2 (18–24 year old females) and Figure 3 (males aged 65 years and over), respectively. Figure 4 displays trends in national apparent alcohol consumption data. Divergent trends in consumption of RTDs and other spirits from 2008/09 are observed.

## Discussion

This study demonstrated a statistically and practically significant increased trend in the rate of acute alcohol ED presentations among 18–24 year old females following the introduction of the GST. Taking into account the estimated changes in slope and level, there were at least 3 260 additional ED presentations by 18–24 year old females during the 94 months from 1 July 2000 to 30 April 2008.

Following the alcopops tax, there were statistically significant decreased trends in the rate of acute alcohol ED presentations among males and females aged 15–50 years, and females aged 15–65 years. The greatest change was in 18–24 year old females with at least 1 350 presentations avoided during the subsequent 44 months, followed by 18–24 year old males with 514 presentations avoided.

Strengths of the study include: a long time series; adjustment for potentially confounding economic factors; use of a large database of ED presentations including both urban and rural populations and a conservative approach to estimating the impact of the taxation measures.

The *slope* changes estimated in our model represent a far more substantial cumulative effect over the longer term than the constant shifts in *level*. The slope estimates represent a monthly rate of change which builds over time; consistent with a gradual change in alcohol consumption behaviour driven by adaptive responses to tax changes by both consumers and the alcohol industry. The negative constant shifts can be interpreted as an artefact of fitting straight-line segments to the data.

Introduction of non-linear methods for controlling for autocorrelation, such as spline terms, and using Poisson regression may have improved model fit and provided better control of residual autocorrelation. However, our aim in this study was to use a sound statistical approach that was readily interpretable and which used a method that may be more familiar to policy makers, who are an important audience of this work.

The results from this study differ from previous Australian studies that found no change in harm associated with the alcopops tax. However these studies analysed trends within much shorter time periods before and after the introduction of the tax compared with this study [14-16].

Surveys of alcohol consumption conducted before and after the introduction of an alcopops tax in Germany suggested a post-tax substitution effect [17]. Australian data indicate that there has been a partial substitution effect post alcopops tax, particularly to straight spirits (Figure 4) [12] and cider [18]. Authors of the German study and others have argued that alcohol related harm in a population is more related to net alcohol consumption than beverage-specific consumption [5,19,20]. Nevertheless, this study, which directly measured alcohol-related harm, indicated a net reduction in harm especially among young women who are a primary marketing target for RTDs. Drink substitution post alcopops tax, as described above, may explain why an even greater reduction was not observed.

The alcopops tax was introduced in Australia during the period of the GFC. A change in alcohol consumption patterns associated with the GFC has been described in other countries [21]. In NSW, overall retail turnover of alcohol continued at similar levels from 2008 onwards (Figure 1). Including liquor retail sales data in our model effectively controlled for seasonality and long-term trend as confirmed by model diagnostics. Our conclusions are therefore conservative as we only found residual changes in harm beyond those explained by trends in alcohol retail sales. A limitation of the data is that it does not account for liquor purchased in bars, clubs or restaurants [10].

A range of alcohol policy measures, such as legislation on liquor licensing, random breath testing and responsible service of alcohol were in existence, introduced or modified over the study period. These may have explained some variation observed over certain time periods. While analysing the complex interactions among these was beyond the scope of this study, policy measures that were likely to be confounders in our study were closely examined. The NSW 2007 Liquor Act, which came into effect in July 2008 was identified as a potential confounder. However, the main effect of the Act was to reform the process and fees for obtaining liquor licenses to ‘promote greater ease and flexibility in establishing a drinking venue.’ [22] It is therefore unlikely to explain the study findings.

Over the study period broader social and cultural factors like employment, migration and urbanisation may have influenced people’s relationship with alcohol in NSW. In this study it was not possible to assess the extent to which such factors may have explained our study findings.

A limitation of this study is that the absolute number and population rate of acute alcohol-related ED presentations in NSW is substantially underestimated by the 39 hospitals. Nevertheless, the hospitals accounted for over one half (57%) of public hospital ED activity and serve most Sydney metropolitan and major regional areas, including key entertainment districts. Apart from some reorganisation of services, the number of public hospital EDs did not change during the study. The constant pattern of alcohol ED presentations in older age groups suggests geographic patterns in drinking did not vary substantially and therefore was unlikely to explain trends observed in young people.

A further limitation is that alcohol ED diagnoses under-estimate the alcohol burden on EDs [23]. However, they do provide a specific marker of alcohol harm that enables the measurement of trends in harm over time. ED information systems in NSW provide diagnoses recorded by clinical or clerical personnel, not by trained coders therefore inconsistencies can occur. In particular, the gradual introduction of SNOMED CT for diagnosis coding along with new patient management software from 2007 in NSW may have led to changes in ED diagnostic recording. The constant trend observed in older patients suggests this was not a major concern. Further, the observed trends between 2000 and 2008 were consistent with the findings from two studies from Victoria during a comparable period [24,25].

There was no ideal control group available for this study. Geographical controls were not possible to obtain as the taxation measures were national. This means that local potential confounding factors, could have caused the trend observed in NSW. However, similar results from Victoria suggest that the change in trends in alcohol harm was not a NSW specific effect [24,25]. In this study, an older age group for whom RTDs are not a preferred drink [26] were used as a control group. The study observed the strongest alcopops tax associated decline among young adults and under-age drinkers, consistent with higher profile of RTDs in that age group thereby providing reassurance of an RTD-specific effect.

The results of this study support international evidence on the effectiveness of alcohol taxation in reducing harm. In Australia, modelling has shown that volumetric taxation, that is, applying the same rate of tax per litre of alcohol across all beverages, would be most efficient at reducing alcohol harm [27]. The results of this study support the notion that selective alcohol taxation may have differential effects on subpopulations, hence a tiered taxation system may be preferred to volumetric taxation where reducing harms in specific subpopulations is the policy objective. This is an important consideration for alcohol policy makers in Australia and globally.

## Conclusion

This study analysed the impact of two taxation measures, with opposite effects on the price of RTDs, on alcohol-related harm seen in emergency departments. Trends in ED presentations for acute alcohol problems in younger people, especially young women, were inversely correlated with the direction of the price change. The findings show that the 2008 alcopops tax coincided with a reduction in alcohol related harm among younger persons, and a partial reversal of the strong upward trend in harm in young women that followed the introduction of the GST. The results of this study support the use of alcohol taxation as an instrument to reduce alcohol harm.
